# A Qualitative Evaluation of Adverse Drug Reaction Reporting System in Pakistan: Findings from the Nurses’ Perspective

**DOI:** 10.3390/ijerph17093039

**Published:** 2020-04-27

**Authors:** Rabia Hussain, Mohamed Azmi Hassali, Anees ur Rehman, Jaya Muneswarao, Muhammad Atif, Zaheer-Ud-Din Babar

**Affiliations:** 1 Department of Social and Administrative Pharmacy, School of Pharmaceutical Sciences, Universiti Sains Malaysia, Penang 11800, Malaysia; azmihassali@usm.my (M.A.H.); aneesurrehman@bzu.edu.pk (A.u.R.); jayamrao@student.usm.my (J.M.); 2 Department of Pharmacy, The Islamia University of Bahawalpur, Bahawalpur 63100, Pakistan; muhammad.atif@iub.edu.pk; 3 Department of Pharmacy, University of Huddersfield, Huddersfield HD1 3DH, UK; z.babar@hud.ac.uk

**Keywords:** nurses, pharmacovigilance, Pakistan, adverse drug reaction, qualitative interview, ADR reporting system

## Abstract

The contribution of all key healthcare professionals is vital to promote an efficient adverse drug reaction (ADR) reporting system. In this context, nurses are important as they are in a better position to observe a patient’s response regarding the drug therapy and to report an ADR. The aim of the study was to explore the perspectives of nurses about ADR reporting system in Lahore, Pakistan. A total of 21 nurses were interviewed. The thematic content analysis of the qualitative interviews yielded six major themes and eight subthemes. Major themes included: (1) Knowledge about the concept of the medication safety & the ADR; (2) Knowledge regarding pharmacovigilance activities; (3) Willingness to report; (4) Practices related to the ADR reporting; (5) Barriers to the ADR reporting; (6) Facilitators to the ADR reporting. The majority of the nurses were aware of medicine safety and ADRs, but in many cases, they were unable to report these ADRs. The study pointed out considerable concerns regarding the knowledge and practices of nurses about pharmacovigilance activities in their workplace, mainly due to increased workload, due to the absence of a reporting system and legal liability. The main challenges turned out to be the lack of knowledge and training, as well as the implementation of guidelines. Based on the findings, it is suggested that outcome of this study can serve as a guide to design policies that support ADR reporting by nurses in Pakistan.

## 1. Introduction

Saving the patients from the perceived harm of medicines and to improve public health, the development of a mechanism is crucial. This mechanism can help evaluate and monitor medicine safety in clinical use. The system of improving medicine safety is known as “pharmacovigilance” which is an umbrella term, comprises of effective drug regulation systems, public health programme and clinical practice to describe the process for adverse drug reaction (ADR) monitoring and its evaluation [1]. A comprehensive and efficient PV system not only considers the identification of risks in data collection, but also takes into account risk evaluation, its minimization and the communication of risks, thus saving the population from the harmful effects of drugs. This is done through a structured system of post marketing surveillance by making appropriate decisions to improve the safe use of medicines [2]. Post marketing surveillance is concerned with the techniques for the detection and measurement of the incidence of ADRs. It refers to the analysis of data collected for the purpose of detecting adverse effects after a drug receives marketing approval [3].

It is considered that a contribution of all multidisciplinary healthcare professionals is key to successful post-marketing surveillance. In this context, nurses are important as they are in a better position to observe a patient’s response regarding drug therapy and thus can report any ADR [4,5]. It has been observed in some studies that contribution of nurses towards the quality and quantity of the reported ADRs were noticeable, and even the quality of the reports was found similar to what physicians reported [6,7,8]. However, their contributions to the pharmacovigilance system are still very limited in many developing countries, including Pakistan [9].

Pakistan is a lower middle-income country with a population of 207.8 million and ranked as the fifth most populous country in the world [10]. There are 195,896 physicians, 99,228 nurses and 32,511 pharmacists in Pakistan’s public sector [11]. Nursing in Pakistan is categorized into general nursing, public health nursing, and midwifery. All three cadres of nursing are registered under Pakistan Nursing Council as registered nurses (RN), lady health visitor (LHV), and registered midwives (RMs) [12,13]. Nurses are usually deployed in hospital settings, while LHVs and RMs are confined to the community for the provision of child and maternal healthcare. Pakistan stands among those countries, where the nurse to doctor ratio is 1: 2.7 and this low ratio heavily compromises the role of a nurse in the healthcare setting [14]. In Pakistan, nurses in a hospital setting, are responsible for carrying out functions like the distribution of medicines, carrying out orders for doctors, and performing other administrative functions [15].

Nurses are key healthcare professionals in any health system, and their roles and opinions are important to develop evidence informed policies [16]. Hence, in this context, the present study assesses adverse drug reaction and it’s reporting related knowledge, attitude, and practices of nurses by employing a qualitative approach. To our knowledge, this is the first study of its kind in the Pakistani context.

## 2. Methods

### 2.1. Ethical Approval

The study was approved under reference no. HEC/PUCP/1943 by the Humans Ethics Committee (HEC), University College of Pharmacy, University of the Punjab, Lahore, Pakistan.

### 2.2. Study Design

A qualitative research approach was selected, as it serves two main purposes. Firstly, it provides a better understanding of the individuals and their relevant experiences, thus producing the rich contextual data, based on experiences, beliefs, and observations. Secondly, it identifies the gap that would otherwise remain unnoticed during a quantitative research design [17,18].

### 2.3. Study Sampling 

Sampling in a qualitative study is based on non-probability sampling. The sampling strategy does not have any published guidelines and it is usually based on the purpose of the research project. For the qualitative study, the required sample size is estimated to reach a point, where no more new information is coming out and this point is known as saturation point [19]. Saturation is considered as the construction of theoretical aspects of inquiry. It relates to the building of rich data within the process of inquiry, by attending to scope and replication. While the scope of data means the comprehensiveness and the depth of the topic covered in qualitative research, replication means data having common but essential characteristics from several participants [20].

The targeted participants were recruited from five tertiary care public hospitals of Lahore, with the approval from the nursing heads. The selected participants were registered nurses from Pakistan Nursing Council and working as full-time permanent employees. They were purposively selected and their convenience as per time and place availability with prior experience and an understanding of the health systems was considered as the factor of their inclusion in this study [21,22]. All the nurses voluntarily participated in this study and no incentive was offered to them to participate in the study.

### 2.4. Data Collection

**Figure 1:**
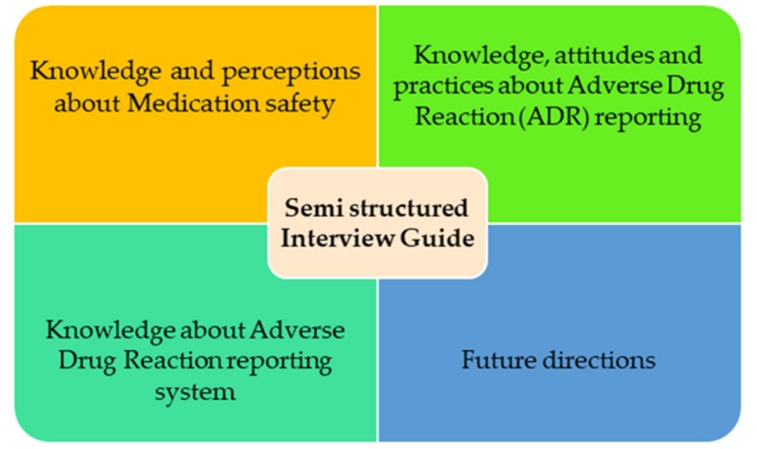
Summary of the topic guide for the semi-structured interview.

The qualitative interview approach was adopted to collect a data rich in individual’s experience. Thus, relating to the topic concerned, semi-structured interviews were considered as appropriate. Semi-structured interviews are based on the interview guide which has predetermined topics or questions but loose in structuring the topic/issues to be explored during the interview. Such interviews have a flexible agenda to follow and the guide give the freedom to the researcher to interview several people in a more systematic manner [23]. A semi-structured interview guide (Supplementary Materials) was developed based on an in-depth review of the literature and by considering current practices of nurses in Pakistan [4,6,7,24,25,26,27,28,29,30,31,32,33]. A summary of the topic guides for the semi-structured interview is given in Figure 1.

An explanatory statement, detailing the study objectives, was given to each participant, and a written consent was obtained from them. The personal information of the participants was taken on a data collection form. The principal author of the study interviewed the participants at their workplace between December 2018 and March 2019. The interviews were conducted in the English language, and each interview lasted for 30–40 minutes. Appropriate probing questions were asked where necessary to seek more information. All interviews were audio-recorded and transcribed verbatim. Data saturation was achieved after 19 interviews. However, two additional interviews were conducted to see if any new themes were emerging.

### 2.5. Data Analysis

The data were analyzed manually by the reading and re-reading of the interviews, and an inductive and flexible approach was undertaken by the research team [34]. The assigned co-authors read interview transcripts to confirm that the generated codes and themes were truly reflective of the content of the interviews, and a mutual consensus was reached among all assigned research team members. A summary of the different phases of analysis is presented in Table 1.

### 2.6. Reporting

The COREQ (consolidated criteria for reporting qualitative research) checklist for reporting qualitative studies was considered for the reporting of the methods and results [35].

## 3. Results

### 3.1. Demographic Details of the Participants

**Figure 2:**
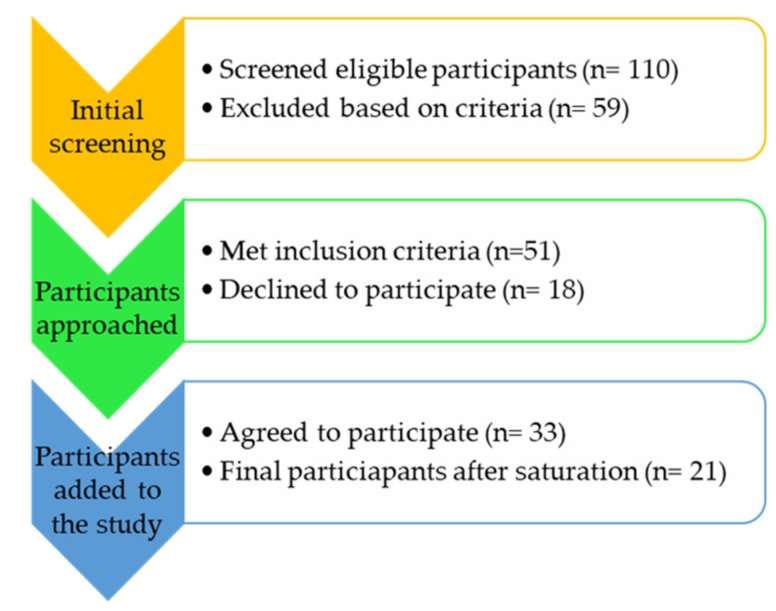
A flow diagram of the participants’ recruitment for the qualitative interviews.

A total of 21 nurses aged between 20 and 40 years were interviewed. A flow diagram of the participants’ recruitment is given in Figure 2.

All participants were female nurses. The majority of the participants (n = 12) were less than 30 years of age, while the rest were below 40. Of all, 13 participants had an experience of less than 10 years, while eight nurses had an experience of 15 or more years of service. Six participants had basic diploma of nursing, while fifteen participants were graduates with a specialized nursing degree known as Post RN (Registered Nurse). The demographic distribution of the participants is described as below (Table 2):

### 3.2. Thematic Analysis of the Content

Thematic content analysis of the interview resulted in six major themes. These themes and subthemes are presented in Table 3 below.

#### 3.2.1. Theme 1: Knowledge about the Concept of the Medication Safety & the ADR

##### Subtheme 1: Knowledge about the Definition of Medicine Safety and ADR

Almost all interviewed nurses gave a detailed response about medication safety concept and it was related to the “five rights of the patient”. A few of them also related this with the medicines that are free from all possible side effects. This shows that almost all nurses were well versed with the concept of medication safety.

“We have five rights of patients, right route, right drug, time, patient, and administration. According to these rules, patient safety can be ensured.”

(N1)

“Medication safety means medicine free of harm.”

(N2)

“Medicine safe from all possible side effects.”

(N13)

When participants were asked about ADR definition, they described an ADR as:

“I know adverse drug reaction, its deviated from the pre-mentioned side effects and they are not expected as such and happen at normal doses.”

(N6)

“ADR is a drug reaction which can endanger the life of a patient at doses which are normally given to the patients to treat.”

(N19)

##### Subtheme 2: Perceptions towards Types of ADR Need to be Reported

Upon asking about types of ADR and the need to be reported, participants gave a mixed response, as some participants thought that major ADRs should be reported, as they could be harmful to the patient’s life. However, others stated that both major and minor ADRs should be reported.

Some stated that the major type of ADRs should be reported, while minor can be treated easily but majority of the effects are life threatening, hence they should be reported.

“I think every type of reaction should be reported. because my personal experience says that we need to be careful and should not ignore even the minor one.”

(N3)

“Major one should be reported, because minor can be tackled but those which cannot be managed, obviously should be reported.”

(N11)

#### 3.2.2. Theme 2: Knowledge Regarding Pharmacovigilance Activities

When nurses were asked about the pharmacovigilance or related activities, they were not aware about the ADR reporting and pharmacovigilance. It was found during the interviews that the majority of the nurses were directed to report any drug related issue to the senior nurse who further conveys this to the in-charge doctor.

“Not at all, no basic guidance means such responsibility have not been assigned to us, we have not been taught this way, we are just supposed to inform the doctors. There is no such activity in my hospital.”

(N11)

“I will tell this to my senior nurse, and she will convey this to the doctor. The rest, I don’t know.”

(N15)

#### 3.2.3. Theme 3: Willingness to Report

The participants in interviews exhibited a positive attitude and showed strong willingness to report of an ADR provided the system allows them to do report them freely. Their views are presented as:

“For me, it’s easy, but due to burden and lack of a system, I cannot report, otherwise its easy and once it’s part of duty.”

(N3)

“Well, simply, its motivation to do something new, to implement and to follow something important as it will improve my practice.”

(N10)

“I am a competent authority, I have knowledge. I know that my voice will be heard, I have experience and I have a duty to report, so I think it’s very easy for me to report an ADR.”

(N17)

#### 3.2.4. Theme 4: Practices Related to ADR Reporting

The nurses were asked regarding the procedure of reporting of an ADR in their hospital setting, like when, how and to whom they are supposed to report an ADR. A mixed practice was observed, where few participants were aware about ADR reporting form, while others were completely unaware about the existence of the ADR reporting system. Their experiences of ADR reporting are given below:

“Yes, we have basic guidance, in that if a reaction happens, we have to inform the pharmacist by filling the ADR form. So, basic guidance we do have. The rest, we do not know.”

(N2)

“I did not have any, because I never saw any system here to help us in reporting. So, we discuss this verbally. We report to doctors and pharmacists verbally. If they need anything written, we provide them. But usually, we inform this to pharmacist.”

(N5)

“I just wrote with red pen and resume my whole scenario with blue pen. Actually, I have a responsibility just to document, because nobody tells how to highlight it.”

(N17)

“I have seen one in my training. We report to doctor but do not report in a systematic manner.”

(N21)

#### 3.2.5. Theme 5: Barriers to the ADR Reporting

The nurses were asked to express the barriers experienced during ADR reporting. They pointed to a lack of time, lack of a proper Adverse Drug Reaction Reporting System, and the legal liability as the major barriers, and this perhaps interferes with their ADR reporting.

##### Subtheme 1: Lack of Time to Report an ADR

The nurses reported that in the public healthcare setting, mostly they are overly occupied with the incoming patients. Thus, due to increased patient population and fewer staff, they remain busy in attending patients, and do not have sufficient time to report an ADR.

“Here, we have lot of patients, so the main problem is patient load. The willingness is there, and once a system is established, then we can do the reporting.”

(N3)

“For me, it’s easy, but due to the burden and lack of a system, I cannot report. Otherwise, its easy and once it’s part of duty, everybody will follow that.”

(N19)

“Most importantly, the workload is too much for us. This is a major burden. More patients, fewer staff.”

(N21)

##### Subtheme 2: Lack of a Proper Reporting System for the ADRs

The majority of the participants identified the lack of a proper reporting system for ADRs as a major barrier in reporting. Most of the participants were aware and motivated to report as they considered reporting of an ADR as the professional responsibility. However, a lack of a proper system was also considered as a barrier towards their willingness to report.

“Here, we do not have system, but if a system is here, then we can report. It’s our professional obligation, to save the patient from the adverse drug reaction.”

(N13)

“I think system is the main reason, we do not have system. These systems should be there and implemented from top management, SOPs should be there, and every department should be informed about the reporting guidelines, and the reporting should involve healthcare professionals and patient or their caregivers. Lack of training is not as such, if there is no system, there’s no training.”

(N16)

##### Subtheme 3: Legal Liability

When asked about the barriers related to ADR reporting, some participants pointed out legal liability as the barrier, while others did not mention this as a barrier. Some participants thought that reporting save the healthcare provider from many problems, while some had the view that it could impact their jobs. They expressed their views as:

“No, legal liability is not there, until we did administer the right drug.”

(N4)

“Well, reporting saves us from legal consequences. Rather, it sometimes helps us to see the mistakes in administration as we attended, noted, and mentioned the things. So, it’s a good thing rather.”

(N7)

“Legal consequences matter as they will affect your job.”

(N19)

#### 3.2.6. Theme 6: Facilitators to the ADR Reporting

A part of the interview focused on the facilitating factors which can help improving the ADR reporting. The participants suggested several factors that can help in this context. Below are some of the details:

##### Subtheme 1: Incentives

During the interview, majority participants favored that incentives may serve as a motivational factor for reporting of ADR, while some said that incentives do not matter in case of reporting an ADR.

“Nurses do not go for monetary incentives. It depends upon awareness only.”

(N6)

“Yes, job incentives will also motivate, but if the system is proper, genuine reporting will be there.”

(N14)

##### Subtheme 2: The Need of an Online System

During the interview, nurses emphasized that an online reporting system should be in place, as they were not satisfied with the traditional paper-based system of reporting.

“It should. Medication should be online. A paper system is very unethical. I’ve never gone for it. But, unfortunately, we only have papers system. Electronic medication report (EMR) has been used and it has its own limitation.”

(N7)

“A possible suggestion is that there must be a system for ADR reporting even at minor level.”

(N18)

##### Subtheme 3: Availability of Pharmacist

The interviewees were convinced that presence of a pharmacist in the wards can help to improve the safe use of medicines. They also discussed that a pharmacist could train them well in this context.

“I think clinical pharmacist should be employed, so we need them in wards to work with us. If they are there, then we will not be confused, and we can ask them if we experience any problem.”

(N4)

“We need good pharmacist, so that they can train us.”

(N11)

## 4. Discussion

To the best of our knowledge, this is the first-ever qualitative study being done on Pakistani setting regarding nurses’ knowledge, attitude, and practices about pharmacovigilance activities in tertiary care public hospitals. Pakistan is the fifth most populous country in the world with every growing need of healthcare for its population [36]. This ultimately leads to an increased burden on healthcare and requires a bigger and trained workforce to serve the growing needs of the healthcare system. The nursing profession is all about the extensive care of patients, families, and communities and also in acquiring, maintaining, and recovering the goal of optimal health [14]. 

The present study found that there were gaps in the knowledge of nurses, though participants were well informed about medication safety, it’s importance while dealing with the patients and also about the importance of reporting an ADR and its reporting. Our findings were similar to the other studies in other countries, where nurses knew the importance of an ADR reporting, but were least aware of the existence and functions of the PV system [26,33]. Traditionally, nurses have been educated and trained about the five rights of medication administration, including the right medication, right dose, right route, right time, and administering to the right patient. However, due to the limited guidance being offered by five rights of medication administration, it needs extra effort in terms of critical thinking on the nurse’s behalf to tackle the complexity of medication management [37]. The issue might be resolved by considering a major revamp of the nursing syllabus so that they can compete in the field globally [38]. 

The Pakistan Nursing Council (PNC) was established in 1948 as an autonomous, regulatory body and was constituted under the Pakistan Nursing Act (1952 and 1973). It regulates several diplomas and degree programs for nurses throughout the country [12]. Among approved nursing degree programs of PNC, the Post RN-Bachelor of Science in Nursing (BSN) degree is considered as the advanced level degree for nursing [38]. It was first introduced in Pakistan in 1988 by Agha Khan University School of Nursing and Midwifery, and since then, hundreds of students have graduated. However, it is not mandatory for nurses to have a post-RN degree practice [39]. It is evident from the literature that the nurse’s role is important in any healthcare system as they are responsible for the delivery of the most appropriate medicines to the patients [27,37,40,41]. Thus, taking into account the crucial role that a nurse can play in the management of the safety of the patient, there is a need to design an effective updated curriculum for nurses. This initiative might also helpful so that nurses can serve in the field with updated knowledge and skills [38].

In our study, we found that nurses exhibited a very positive attitude towards reporting. Like other studies, the participants in our study were willing to report an ADR provided they are encouraged to report [32]. However, sometimes, they were reluctant, especially when they were not clear about their roles in reporting of an ADR. This was also observed in a study by De Angelis in 2015, where majority of the Italian nurses were willing to report however, being unaware about their own autonomy in the reporting of an ADR, they chose not to report [26]. 

Our study found that, in some cases, nurses, when they were aware that an ADR had happened, usually kept quiet or passed the information to their senior nurses. A study from northern Nigeria has shown that about 42.7% of the nurses have reported 75% of suspected ADRs through verbal communication [42]. It was also observed that in many cases that nurses tend to inform their senior nurses or physicians about ADR and leave them with the decision of reporting [8,28,43]. In Pakistan, like most clinical settings, dependence on a senior is a common practice. Healthcare professionals, especially doctors and nurses, rely on their seniors for the decision-making [44]. The practice is really useful when teamwork is needed. However, when someone is reluctant to speak, this threatens the safety of the patients [45]. Nurses are considered responsible for the prevention, treatment, surveillance, and documentation of drug-related allergies and ADRs. Due to their knowledge and having close involvement in patient’s medication administration, they are in a unique position to detect ADRs and identify early signs of a drug-related problem [46,47]. 

In Pakistan, the National Pharmacovigilance Centre of Pakistan (NPC) was established under the Drug Regulatory Authority of Pakistan (DRAP) Act 2012 as a national center. The NPC was responsible for the liaison between the country’s regional pharmacovigilance centers and with the international pharmacovigilance center [48]. In due course, National Pharmacovigilance Centre became active and started training and publishing a monthly newsletter to inform the healthcare professionals and drug manufacturers regarding the reported ADRs in other countries. DRAP also launched an online reporting form for the computerized reporting of an ADR [48]. However, it came out during a discussion with the participants that a few nurses were reporting ADRs on prescribed forms for ADR reporting, while some participants had no idea about such reporting. They were just performing the traditional roles of a nurse and thought that direct reporting of an ADR is not in their job description. This is very similar to a study by Dilles in 2011, where nurses did not report and were unsure about their responsibility [49]. If they encountered any problem with the drug administration, they were supposed to report this information to the senior nurses and the doctors. However, the current system of healthcare in the country allows all healthcare professionals, patients, and their families to report an ADR to the DRAP, but there is no obligation for anyone to report an ADR. This offers a key opportunity for the nurses to develop their roles as ADR advocates [50,51].

The nurses also identified several barriers that impede the smooth reporting of ADRs. They highlighted the barriers to reporting which include work overload, lack of a proper system of reporting and the fear of being liable to an ADR incident. The similar issues are being reported in other studies [49,52].

Being the fifth most populated country in the world, Pakistan requires a trained and large workforce for effective healthcare delivery. The patient-nurse ratio is far below the WHO standards in the country [36]. According to a WHO report, only 0.57 nurses are available in Pakistan for a 1000 population. Similarly, the physician to nurse ratio is also unable to meet the standard number. It should be 1:3 as per the WHO standards, however, the situation is opposite in Pakistan, where the number of doctors is 2.7 per nurse, claiming the lowest ratio of doctors to nurse and population to nurses elsewhere, thus contributing massive workload for nurses [14,53]. 

Nurses in our study have also identified a lack of an online reporting system as a problem of under-reporting. In Pakistan, few private modern hospitals have drug information and drug safety centers. The practices in these hospitals are world-class and these practices vary from the public sector [54,55]. Most healthcare professionals in these few private hospitals are trained to report, while in most of the public setting, the scenario is equally opposite [56]. This also shows that even in public healthcare settings, if the healthcare professionals are trained and they are provided with a proper system, they can perform their duties very well. Moreover, having an efficient and updated system, time can be saved, and healthcare professionals can perform their duties very well. In the past, only a few studies have been conducted in the country, highlighting the importance of a proper ADR monitoring and reporting system [21,57,58]. However, as Pakistan is now the full member of the WHO, things may improve in regard to the monitoring and reporting of adverse events [59].

Nurses are important healthcare professionals in improving patient safety as they are continuously present at the bedside of the patient and have interactions with the family members and with the other healthcare professionals [60]. The nurses in our study have also highlighted that, if they report an ADR, they will be held responsible on legal terms... This has been found out in many other studies, where participants were afraid to report because of the fear of legal liability [8,26,29,32,43,55]. This problem could be solved if there is a positive culture for ADR reporting in the system which develops as a result of the promotion of patient safety. Such a system could be built on the basis of having a reliable and safe environment and this is usually based on transparency, trust, and accountability maintained by the staff. Ultimately, such a system, if promoted, would reduce errors and would improve the quality of care [61]. As a result of this, the nurses can learn from their mistakes and can be vigilant and more open to speak and report, thus ensuring patient safety in the long run [62,63]. The studies have also indicated that with the promotion of a patient safety culture promotion, there were fewer patients related complications and fewer adverse events [64,65]. A Swedish study has also emphasized that nurses in their daily practice are in a better position to detect and report ADRs. Therefore, nurses should be trusted with a greater responsibility of ADR reporting [66].

Pakistan can learn from good global examples from both the developed and developing countries. The establishment of an online ADR reporting system, the involvement of nurses in the reporting process were suggested as the factors which would contribute to improving the standards of ADR reporting. The nurses in our study did mention the role of pharmacists in improving medicines safety and pharmacovigilance and the support they can provide to nurses. This has been also mentioned in other studies where it was stated that the role of the pharmacist is vital to improve overall medicines safety and has resulted in increased learning regarding pharmacovigilance among hospital staff including nurses. In 2007, Costello also studied the effects of a pharmacist-led learning on medication error reporting in pediatrics and found an increase in medication error reporting [67]. Similarly, a cluster-randomized trial of pharmacist partnering in medication management showed a decreased number of medication errors within the first 24 hours of hospital admission [68]. A meta-analysis of pharmacist-based interventions in 2017 by De also noted a significant reduction in hospital emergency visits after the patients discharge and 37% of the decrease in medication errors [69]. This also shows the positive impact of the pharmacists to improve the concept of medication safety.

To overcome problems in the knowledge and skills of nurses, educational programs and training regarding pharmacovigilance can be introduced in all tertiary care public hospitals in Pakistan. Examples can be drawn from global studies, indicating the need of ADR related training and awareness-based programs for nursing staff [70]. Dilles in 2010 also pointed out that the nurses with higher qualifications were more involved in the identification of ADRs, when compared with their counterparts [71]. Similarly, a study by Valente & Murray in 2011 indicated that a nurse-led educational intervention not only resulted in the increased knowledge for nurses, but also led to an increased reporting of ADRs by the nurses [47]. In 2012, Conforti concluded that the nurses’ participation in Italian regional pharmacovigilance centers significantly improved the reporting standards and the resultant outcomes [25].

### Strengths and Limitations

A possible limitation of our study is that the nurses participated in this interview may have given responses intended to express socially desirable views during the interviews. However, the participants were assured that their anonymity and confidentiality would be maintained, and it was evident during the interviews that the participants expressed their opinions freely and honestly. Another possible limitation could be that all participants were females. This is due to the fact that in all Pakistani public hospitals, male nurses are not officially recruited and only females serve as nurse in the public sector. Therefore, there is a possibility that male nurses might have different responses. However, the study was conducted in the second largest city, Lahore with more aware nurses. Thus, despite these limitations, the information is still useful for both healthcare professionals as well as for policymakers.

## 5. Conclusions

The findings from our study support the view that the nurses do understand the concept of ADR, but in many cases, they were unable to report it. This was due to barriers related to high workload, lack of ADR reporting system and prone to have a legal liability. Although, the nurses were agreed to report ADRs, but they should be adequately trained to detect, monitor and report ADR. In this regard, the current nursing curriculum and training modules should be revised as per the international standards. Besides, the hospitals should consider establishing training programs for nurses and other staff under the supervision of pharmacists.

## Figures and Tables

**Table 1:** Data analysis process.

Phase of Analysis.	Tasks Completed	Research Team Member Involved
Phase 1: Data familiarization	Transcription, reading and re-reading of interview transcripts.	RH
Phase 2: Initial codes generation	Initial, open coding of entire data set	RH and MAH
Phase 3: Search for themes	Categorization of codes into potential themes	RH and MAH
Phase 4: Review of themes	Confirming themes—ensuring the internal homogeneity and external heterogeneity of themes.	RH, discussed with MAH.
Phase 5: Defining and naming themes	Further refinement of themes	RH, confirmed with MAH.
Phase 6: Report finalization	Production of the manuscript, selection of illustrative quotes	RH, reviewed by and discussed with MAH and ZB.

**Table 2:** Demographics of the participants.

Characteristics	Frequency
Gender	
Male	0
Females	21
Age (Years)	
20–30	12
31–40	9
Education	
Basic nursing	6
Specialization	15
Experience (Years)	
1–5	2
6–10	11
>10	8
ADR reporting	
Yes	4
No	17

**Table 3:** Description of themes and subthemes.

Category	Themes and Subthemes
Knowledge	› Knowledge about the Concept of the Medication Safety & the ADR Subtheme 1: Knowledge about the definition of medicine safety and ADR Subtheme 2: Perceptions Towards Types of ADR Need to be Reported › Knowledge Regarding Pharmacovigilance Activities
Attitudes	› Willingness to report
Practices	› Practices related to the ADR reporting
Barriers	› Barriers to the ADR reporting Subtheme 1: Lack of Time to Report an ADR Subtheme 2: Lack of a Proper Reporting System for the ADRs Subtheme 3: Legal Liability
Facilitators	› Facilitators to the ADR reporting Subtheme 1: Incentives Subtheme 2: The Need of an Online System Subtheme 3: Availability of Pharmacist

## Supplementary Materials

The following are available online at https://www.mdpi.com/1660-4601/17/9/3039/s1, Supplementary file S1: Interview Guide.
