# Supplementary material for: A Qualitative Evaluation of Adverse Drug Reaction Reporting System in Pakistan: Findings from the Nurses’ Perspective
Source: Int J Environ Res Public Health. 2020 Apr 27;17(9):3039. doi: 10.3390/ijerph17093039 (PMC7246579; doi:10.3390/ijerph17093039)
Supplement: Supplementary file 1 [file ijerph-17-03039-s001.pdf]

Interview Guide

Objective: Exploring the knowledge & perceptions of nurses towards medication safety & Adverse Drug Reaction (ADR) reporting system

Part I

Focus: Knowledge and perceptions about Medication safety

1. What comes into your mind, when you hear the word "Medication Safety".
2. Do you counsel patients on medication safety? If yes which particular aspect of it, under what conditions do you counsel them and why? (condition mean disease state)
3. Any recent counselling being offered to patients, any examples (prompt for conversation, symptoms, recommendations / advice given)

Part II

Focus: Knowledge and attitudes about Adverse Drug Reaction (ADR) reporting

1. Do you know, what is Adverse Drug Reaction? How would you define it?
2. What would you do if you were approached by a patient with a severe ADR (any recent incidence, what was your strategy to deal with the patient)?
3. What type of adverse drug reactions you consider should be reported?
4. In your organization, who is responsible for ADR reporting?
5. Do you have any guidance on reporting or how to and when to report any ADR? And report to whom and Where?
6. Have you ever sent an adverse drug reaction report to your national reporting agency, when that happened and what is the reason behind?
7. Have you ever sent an adverse drug reaction report to the responsible pharmaceutical company, when that happened and why did you send it?
8. What are the factors that you think can impact and may encourage nurses to report ADRs (why a nurse should report an ADR)?
9. In your opinion, what are the possible factors that contribute as the barriers to ADR reporting?
10. Do you think your job in any way makes it easy/ difficult to report ADR.

Part III

Focus: Knowledge about Adverse Drug Reaction reporting system

1. Are you aware about the existence of the regulatory body that regulates ADR reporting in Pakistan? Reasons? if you are not aware? (education/ training)
2. In your opinion, do you think that there is a need to change the system about medicine safety and ADR reporting? What benefit would it have?

Part IV

Focus: Future perspective

1. Do you think that your role has changed and how it can contribute towards medication safety?
2. What could be the possible suggestions to improve ADR reporting in hospital setting in future?

Conclusion/Suggestions

Would you like to provide any additional comments about medication safety and ADR reporting system in Pakistan?
